# Analysis of the Variations in the Colic Branching Pattern of the Superior Mesenteric Artery: A Cadaveric Study With Proposal to Modify Its Current Anatomical Classification

**DOI:** 10.7759/cureus.25025

**Published:** 2022-05-15

**Authors:** Sobal Nigah, Apurba Patra, Shekhar Chumber

**Affiliations:** 1 Anatomy, Dr. Bhimrao Ramji (BR) Ambedkar State Institute of Medical Sciences, Mohali, IND; 2 Anatomy, All India Institute of Medical Sciences, Bathinda, IND; 3 Forensic Medicine, Gian Sagar Medical College and Hospital, Rajpura, IND

**Keywords:** ileocolic artery, right colic artery, middle colic artery, superior mesenteric artery, colon, anatomic variation

## Abstract

Background: Vascular anatomy of the ascending colon is more complex and variable as compared to the left. Variations range from the mode of origin, branching to territorial supply. The present study was undertaken to learn the anatomical variations of the superior mesenteric artery (SMA) and its colic branches in detail.

Materials and methods: The study included 50 formalin fixed cadavers aged between 40 and 65 years. The colic branches arising from SMA were dissected to trace their mode of origin, branching pattern, and territorial supply. Based on the mode of origin of the colic branches, the SMA anatomy was classified into four patterns: I; II a, b, c; III; and IV.

Results: SMA itself showed variations in its origin (as celiacomesenteric and hepatomesenteric trunk), so the colic branches arising from it. In most of the cases, middle (MCA), right (RCA), and ileocolic artery (ICA) originated independently from SMA (I). A common stem (CS) was reported between MCA and RCA (IIa) in seven cases; RCA and ICA in one (IIb); MCA and LCA in one. MCA originated from the coeliac trunk (CT) in one case. In one case, each of absent RCA (III) and accessory artery arising from SMA (IV) was also noticed. In one case, the right branch of MCA gave origin to RCA. Additionally, close association was observed between pattern IIa and incomplete inter-colic anastomosis.

Conclusion: Variations in the origin of SMA may potentially influence branching patterns of colic arteries. MCA is the most variant and ICA is the most consistent branch of SMA. Distinctive variations like MCA arising from CT or arising as CS with LCA and incomplete inter-colic anastomosis in pattern IIa are of outrageous importance for operating surgeons during surgical procedures of colon. Based on study results, we propose a modification in the classification of SMA anatomy to include the CS of MCA and LCA as type IId; however, its success relies upon universal acceptance.

## Introduction

Superior mesenteric artery (SMA) arising as an unpaired ventral branch of the abdominal aorta is the main artery supplying the midgut, i.e., from the second part of the duodenum where major duodenal papilla opens up to the junction of right two-thirds and left one-third of the transverse colon [1]. The SMA originates from the aorta as the second ventral branch 1 cm below the coeliac trunk (CT), at the level of the intervertebral disc between the first and second lumbar vertebrae. It then runs anteriorly and inferiorly to the uncinate process of the pancreas and the third part of the duodenum. As it descends down through the root of mesentery, the course of the artery is anterior to the inferior vena cava, right ureter, and right psoas major. The caliber of the SMA goes on decreasing as successive branches are given off to the loops of jejunum and ileum, ultimately it ends in a terminal branch which anastomoses with the ileocolic artery (ICA) [2,3]. Most of the reference textbooks of anatomy and surgery documented that SMA gives off the first colic branch as middle colic (MCA) followed by right colic (RCA) and ileocolic artery (ICA) as independent branches from the right side of the artery [4]. Numerous jejunal and ileal branches arise from left side of SMA which supplies the jejunum and ileum respectively [5]. The derivatives of hindgut are supplied by the inferior mesenteric artery (IMA) which is also a ventral branch of the abdominal aorta. The anastomosis between branches of SMA and IMA around the concave margin of the large colon forms single arterial trunk known as inter-colic anastomotic artery. This provides ample collateral circulation when required for planning surgical intervention in this area. Occlusion of the SMA can lead to gangrene of right-sided colon; however, gangrene of left-sided colon is rare after occlusion of IMA as it is dependent on the degree of collateral circulation from the hypogastric and SMA [6]. In literature, it is depicted that the arterial variations of the right colon are more complex and variable as compared with the left colon. Variations in the mode of origin, branching, and territorial supply involving the CT and SMA are more common than those of the IMA [7]. Among the branches of the SMA, MCA has been reported to be the most variant branch and ICA's most consistent branch [6]. Awareness of these and other vascular variations is of clinical significance during various surgeries involving the colon to avoid complications such as intraoperative hemorrhage and colonic ischemia [8]. With such a background, the present work was undertaken to study the variations of colic branches of SMA and correlate the findings clinically.

This article was previously published as a preprint in Research Square: Nigah S, Patra A, Chumber S, Kalyan GS: Analysis of the variations in the colic branching pattern of the superior mesenteric artery: A cadaveric study with proposal to modify its current anatomical classification, February 28, 2022 (https://assets.researchsquare.com/files/rs-1390089/v1/379d49f1-2f8e-48bb-9a0b-a2eab14db88a.pdf?c=1646083704).

## Materials and methods

Study design, selection criterion, and place of study

It was a cross-sectional study carried out on 50 adult formalin fixed cadavers (37 male, 13 female), aged between 40 and 65 years with a mean age of 55.84±7.74 years. Adult age group cadavers of North Indian origin and without any history of abdominal surgery or trauma were studied over a period of three years. The study was carried out in the Department of Anatomy, Government Medical College, Patiala, India, after obtaining approval from the competent authority vide letter no. BFUHS/2K11/p-TH/8179.

Methodology

The abdomens of these cadavers were dissected by a cruciform incision passing through the whole thickness of the anterior abdominal wall. After reflecting the skin flaps, abdominal viscera were carefully removed, and the transverse colon along with the mesentery was lifted superiorly. The oblique attachment of the mesentery of the small intestine was traced on the posterior abdominal wall [2]. Thereafter, the small intestine was turned to the left, a cut was given through the right layer of the mesentery along the line of attachment, and the fat from the mesentery was removed meticulously to expose the SMA in its root and the colic branches arising from the concavity of the artery. These branches were traced from their origin up to their area of supply and anastomosis between them along the concave margin of the colon. The SMA arises from the front of the abdominal aorta about 0.5 cm below the celiac trunk at the level of the intervertebral disc between L1 and L2 vertebrae. Usually, three colic branches, right colic, middle colic, and Ileocolic, arise from the concavity on the right side of the SMA craniocaudal fashion. Most cranially arise MCA, it enters the transverse mesocolon and divides into right and left branches that anastomose with the branches of RCA and left colic artery (LCA) to complete the marginal artery. The anastomosis between MCA and LCA at the level of the marginal artery may be deficient. RCA arises next to MCA; on reaching the ascending colon it divides into ascending and descending branches which anastomose with the branches of MCA and ICA to take part in the formation of the marginal artery. The ICA is actually the continuation of the SMA. It divides into ascending and descending branches. The ascending branch anastomoses with the descending branch of RCA. The descending branch supplies the terminal ileum by its ileal branch, cecum by anterior and posterior caecal arteries, appendix by appendicular branch, and the lower part of ascending colon by colic branch. These colic arteries show variant patterns in their mode of origin, branching, and area of supply. Based on the mode of origin of the colic arteries, the SMA anatomy was classified into four patterns [9]: pattern I - RCA, MCA, and ICA arise independently from the SMA; pattern II - the colic arteries arise as a common stem (CS) or trunk from SMA. Such pattern is subdivided into three sub-patterns based on the common stems or trunks of origin: pattern IIa - CS between RCA and MCA; pattern IIb - CS between RCA and ICA; pattern IIc - CS for the RCA, MCA, and ICA; pattern III - as the absence of RCA; and pattern IV - based on the presence of any additional or accessory artery arising from the concavity of the SMA apart from the colic arteries. The findings were analyzed using descriptive statistics.

## Results

Anatomical variations of the SMA

In most of the cases, it was arising independently from the ventral surface of the abdominal aorta; in four cases (8%), it was sharing its origin with celiac trunk (celiacomesenteric trunk) and in one case (2%), a common hepatic artery takes origin from SMA (hepatomesenteric trunk). When we compared the variations of the colic branches with the variations of the SMA itself, all the SMAs with variant origin showed variations in their colic branching pattern. In one case LCA was arising from SMA instead of IMA. The colic branches of SMA were studied in detail (mode of origin, branches, and area of supply)

Middle colic artery

**Figure 1:**
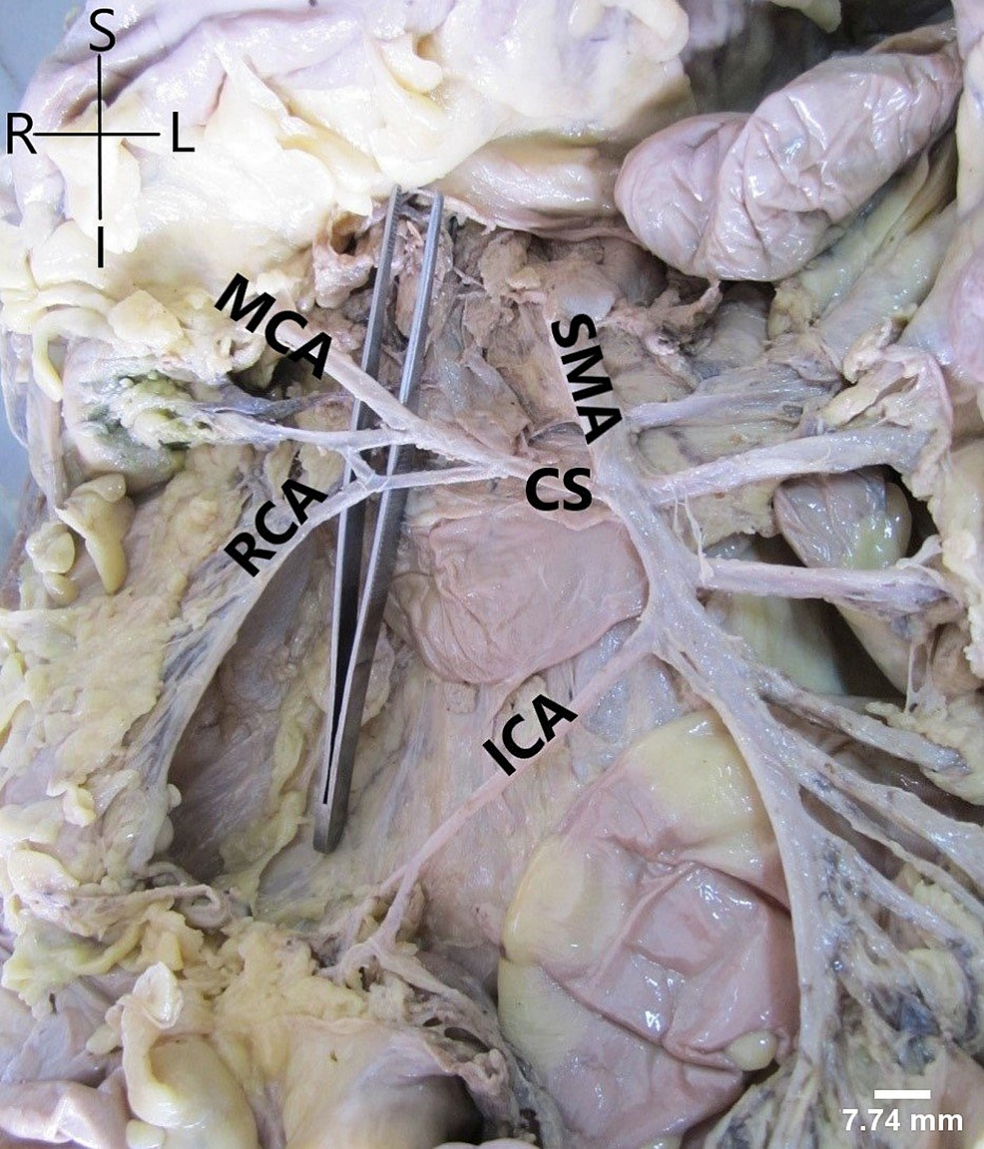
Common stem (CS) of origin of middle colic artery (MCA) and right colic artery (RCA) from superior mesenteric artery (SMA). ICA: ileocolic artery

**Figure 2:**
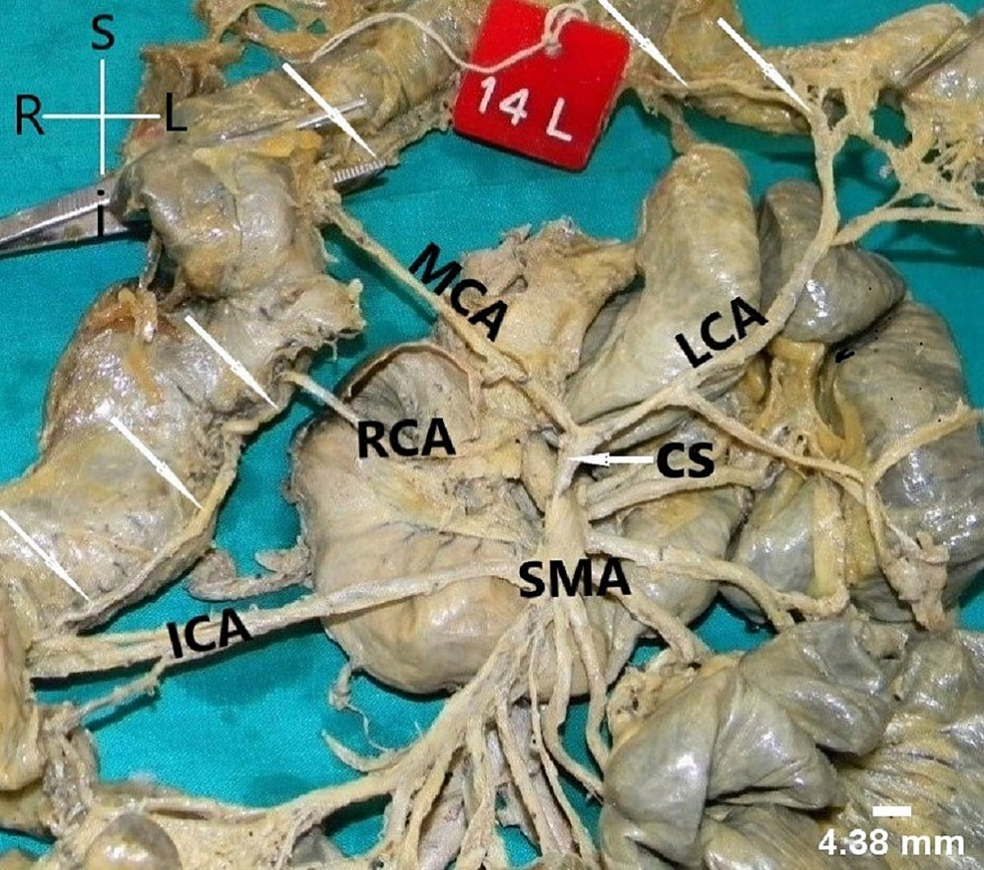
Common stem (CS) of origin of middle colic artery (MCA) and left colic artery (LCA) from superior mesenteric artery (SMA). The arrows show the formation of inter-colic anastomotic artery. RCA: right colic artery; ICA: ileocolic artery

**Figure 3:**
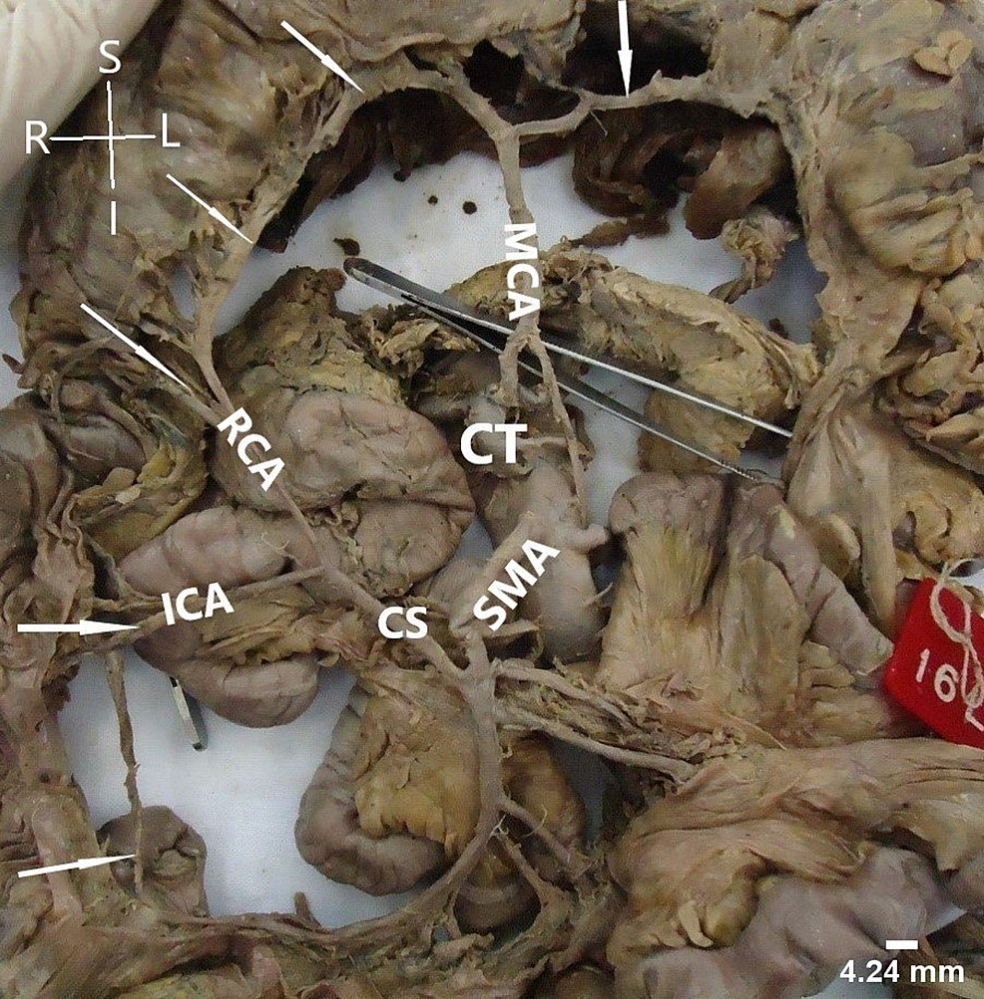
Common stem (CS) of origin of right colic artery (RCA) and ileocolic artery (ICA) from superior mesenteric artery (SMA). The image shows the middle colic artery (MCA) arising from celiac trunk (CT). The arrows show the formation of inter-colic anastomotic artery.

It was found as the first colic branch showing significant and rare variations in its mode of origin and area of supply. In 41 cases, MCA had an independent origin from SMA. In seven cases, it had a CS with RCA (Figure 1). In one case (male), it had a CS with LCA and the LCA was arising from SMA instead of IMA (Figure 2). In one specimen, MCA was found to be arising from CT (Figure 3).

**Figure 4:**
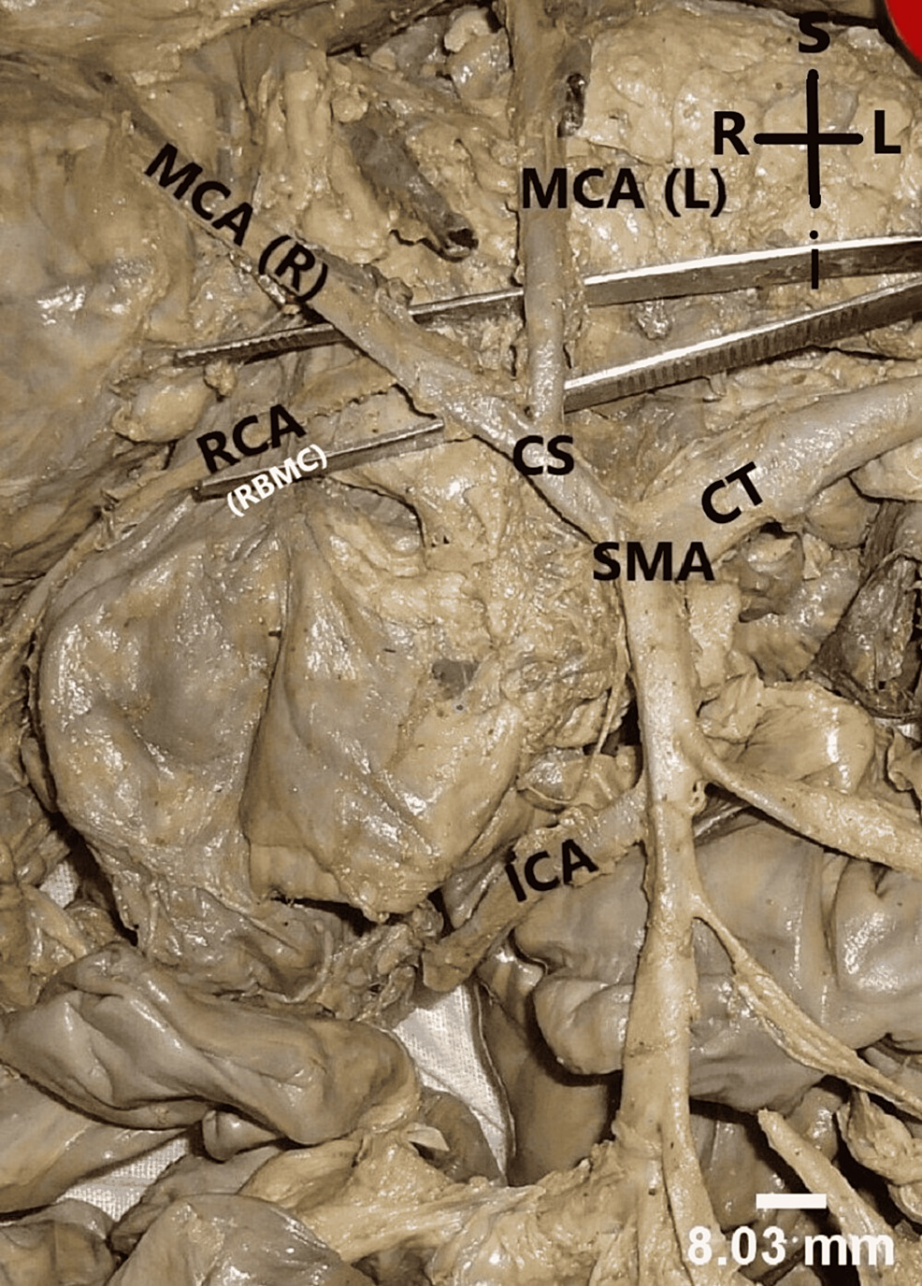
Middle colic artery (MCA) arising from superior mesenteric artery (SMA) bifurcated into right and left branch. The image shows the right branch of MCA was giving origin to RCA (RBMC). The arrows show the formation of inter-colic anastomotic artery.

Irrespective of their origin, MCA bifurcated into right and left branches in all the cases. A significant variation was noticed in one specimen where right branch of MCA was giving origin to RCA (Figure 4). In 48 cases, MCA supplied the right two-thirds of transverse colon. In one case, where RCA was absent, MCA additionally supplied the upper one-third of ascending colon and in another specimen, it was supplying the whole of transverse colon.

Right colic artery

**Figure 5:**
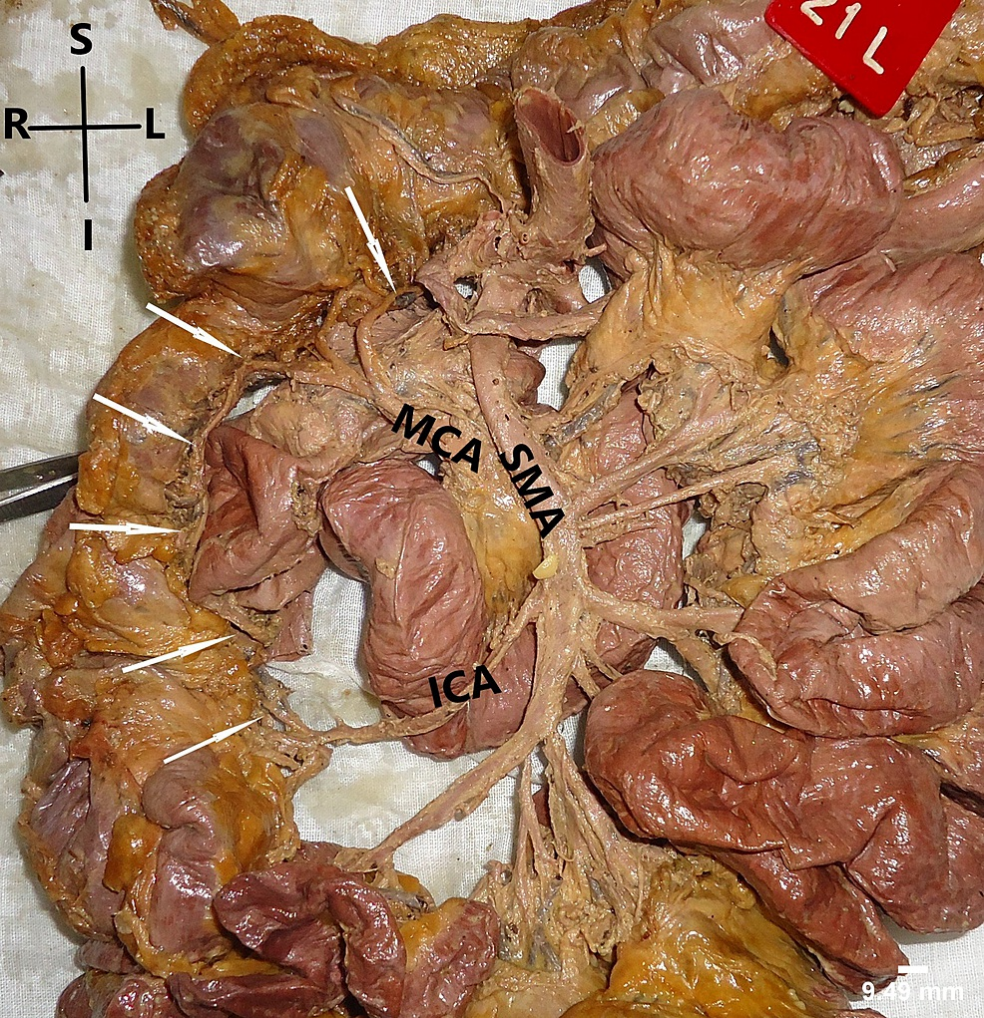
Middle colic (MCA) and ileocolic artery (ICA) arising independently from superior mesenteric artery (SMA). The image shows the right colic artery (RCA) is absent. The arrows show the formation of inter-colic anastomotic artery.

In 40 cases, RCA had independent origin from SMA. In seven cases, it had CS of origin with MCA (Figure 1); in one case, it had a CS with ICA (Figure 3); in one specimen, RCA originated from right branch of MCA (Figure 4); and it was absent in one case (Figure 5).

Branches

In all the cases, RCA was divided into ascending and descending branches except in one case where it arose from the right branch of MCA. In all the cases, branches of RCA supplied the upper two-thirds of the ascending colon including hepatic flexure except in two cases, where RCA was absent and not given ascending branch, the above-said area was supplied by MCA.

Ileocolic artery

Ileocolic artery (ICA) showed the least variations. In all the cases, ICA had an independent origin from SMA except in one where it was arising as CS with RCA (Figure 3). In all the cases, ICA was divided into ascending and descending branches. Ascending branch anastomosed with the descending branch of RCA except in one specimen where RCA was absent; here, it joined the descending branch of MCA. Caecal and appendicular arteries were seen to arise from the descending branch in all the specimens. In all the cases, branches of ICA supplied terminal part of ileum, ileocecal junction, caecum, appendix, and lower one-third of ascending colon.

Accessory arteries (extra colic branches)

**Figure 6:**
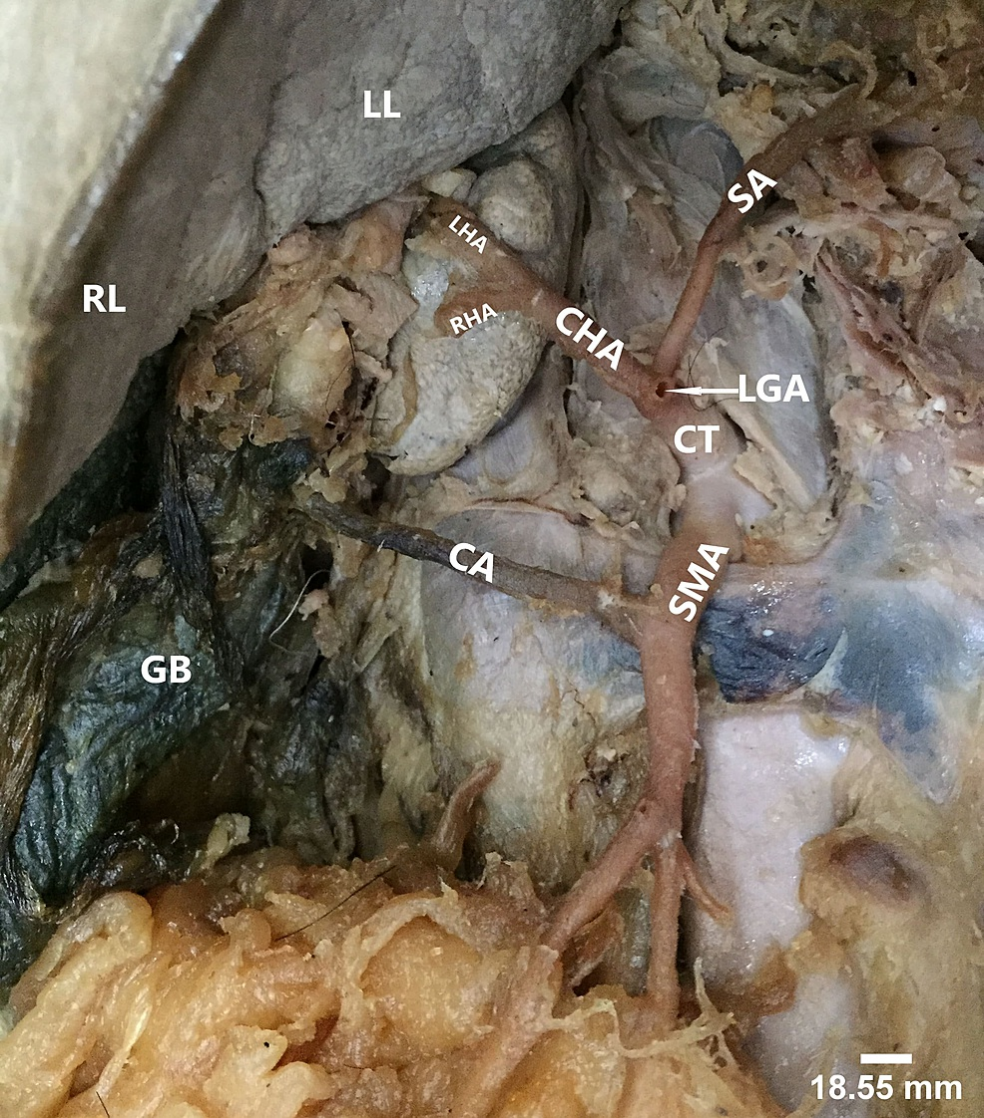
Variant origin of cystic artery (CA) as an independent accessory/extra colic branch of superior mesenteric artery (SMA). The arrows show the formation of inter-colic anastomotic artery. RL: right lobe of liver; LL: left lobe of liver; MCA: middle colic artery; RCA: right colic artery; ICA: ileocolic artery; LCA: left colic artery; IMA: inferior mesenteric artery

In one case, we observed a variant origin of cystic artery arose as an independent extra colic branch from the SMA (Figure 6). Based on Gamo’s classification, this case was typified as pattern IV (presence of accessory arteries) [9].

Patterns of SMA anatomy

Depending upon the mode of origin of colic branches, we found four patterns. Pattern I (independent origin of colic branches) was found most commonly, followed by pattern IIa (CS between MCA and RCA) in seven cases (Figure 1). One case each of pattern IIb (CS between RCA and ICA) (Figure 3), III (absent RCA) (Figure 5), and IV (presence of accessory arteries) (Figure 6) was also reported. We have also reported one case of CS between MCA and LCA and classified the case as pattern IIb (Figure 2).

Inter-colic anastomosis

The anastomosis between colic branches of SMA and IMA around the concave margin of large colon showed variations in their formation. In nine cases, arterial trunk formed by inter-colic anastomosis was incomplete. Out of them, three belonged to pattern I, five to pattern IIa, and one to pattern IV.

## Discussion

Developmentally, the arteries of the gut begin as ventral segmental branches of the dorsal aorta. They are initially distributed to the developing gut and yolk sac as paired primitive vitelline arteries that are more or less segmentally arranged. However, as a result of growth in length and rotation of the gut tube, accompanied by migration of the associated viscera, most of the stems of origin either disappear or fuse with neighboring stems. This results in a reduction in the number of the main arteries to three large unpaired midline vessels, namely, CT, SMA, and IMA supplying the foregut, midgut, and hindgut, respectively. Owing to their closely related embryonic origins, persistence of interconnections or overlapping of the territories of the three main arteries are common. Various authors have described variations that do not conform to the classical patterns of foregut, midgut, and hindgut arterial territories described in standard texts [5,9-12]. Knowledge of these variations is important in order to avoid possible catastrophes during surgical interventions in the regions traversed by these arteries.

In the present study, MCA usually arose as an independent branch from the SMA. However, variations in their mode of origin were also noticed in few instances. In seven cases (14%), MCA originated from SMA in the form of CS with RCA. Sonneland et al. reported the presence of CS of MCA and RCA in 26.70% and 25% of cases, respectively [13]. In one case (2%), we have noticed MCA arise from the CT instead of SMA. There have been very few reports on such variant origin of MCA [14] and the reported incidence of such variation is very low, ranging from 0.5% to 1% [15]. The embryological basis of MCA originating from the CT was considered evidence for the ventral longitudinal anastomosis of the primitive vitelline arteries in the embryo [16]. The precarious origin of MCA from the CT and its dominance in the formation of the marginal artery were thought to predispose the ascending and transverse colon to an increased risk of vascular damage during major abdominal surgery. In one instance, we observed a very rare variation in the origin of MCA where it arose as a CS with left colic artery (LCA) (usually a branch of IMA). The literature reported such variation is very low (0.20%) [17]. As far as the Indian ethnicity is concerned, very few authors have reported single case of LCA arising from MCA, that too independently [18]. Kim et al. also reported single case of LCA arising from MCA in the Korean population [19]. So, what makes our finding interesting is that over here LCA was arising from SMA as a CS with MCA. Gamo et al. classified SMA anatomy based on the mode of origin of colic branches but did not take the CS of MCA and LCA into consideration might be because of its rare occurrence [9]. Based on the study results, we propose modification in Gamo’s classification of SMA anatomy to include the CS of MCA and LCA as pattern IId. Such modification is a simpler and better way to describe SMA anatomy based on its branching pattern, however, its success relies upon universal acceptance.

In the present study, MCA was supplying the right two-thirds of transverse colon almost in all the cases; among them, in one case, an additional area of the upper one-third of ascending colon was also supplied by MCA as RCA was absent. The text of Holinshed surgical anatomy documented that in the absence of RCA, the chief source of arterial supply to the uppermost part of ascending colon and right colic flexure is through branches of MCA [12]. In the specimen, where MCA and LCA arose from the SMA as a CS, the left branch of MCA also supplied the left one-third of the transverse colon which is normally a territory of IMA. Jain and Motwani reported the rarest variation in the branching pattern of SMA, where a CS which soon divided into a larger LCA and a smaller splenic (accessory) artery was found [16]. Thus, the LCA arose from SMA supplied the whole of the transverse colon and upper part of descending colon, which is normally the territory of IMA.

In most of the cases, RCA had independent origin from SMA but variations in mode of origin were found in nine cases. Out of them, in seven cases, it arose as a CS with MCA and in one case it originated from right branch of MCA. Reviewing literature showed that RCA may arise from the root of the MCA or from its right branch. Most of the researchers have reported RCA arising from the root of MCA [17,18,20,21]. While very few people observed RCA arise from its right branch [22].

RCA arising as a branch of ICA is also commonly reported [18,21,22], while RCA shared a CS with ICA reported rarely [17]. In the present study, only one case (2.85%) was observed where RCA was sharing CS with ICA, whereas Jain and Motwani reported such variant mode of origin of RCA in 25% of the study population [16]. Moreover, one case (2.85%) with absent RCA was found, which was in agreement with the findings of Nelson et al. [18]. Systematic review showed that the absence of RCA ranges between 2.00% and 18.00% with weighted mean incidence of 8.9% [20].

Multiple attempts have been made over the decades to provide a unifying definition for the right colic feeding vessel but confusions still persist [22]. According to surgical context, it was regarded as any artery supplying the hepatic flexure of the colon; however, no consideration was given to its origin [17]. In recent days, some researchers have proposed that the term "right colic artery" be reserved for a vessel arising independently from the SMA, while "right colic branch" be applied in all other cases [19]. This proposal was supported by the systematic review done by Haywood et al. owing to the high frequency with which the RCA arises from a vessel other than the SMA [4].

Determining the exact level of bifurcation, origin of the right colic feeding vessel and precise understanding of the relevant anatomy is crucial to perform more advanced surgical interventions effectively [13,23,24]. Haywood et al. systematically reviewed 10 studies involving 1073 cadavers to determine the mean incidence with which the RCA arose from other parent vessels and reported that to be 36.80% for the SMA, 31.90% for the ICA, 27.70% for the root of the MCA, and 2.5% for the right branch of the MCA [22]. In 1.10% of individuals, the RCA shared a trunk with the MCA and ICA and in 8.90% of cadavers, the RCA was absent. Only a few smaller data series described the right branch of MCA (RBMC) as a potential origin of the RCA, resulting in a low mean incidence for this particular arrangement as in our study [1,18]. So, the origin of the right colic feeding vessel is highly variable, arising from the SMA, ILC, MCA root, and RBMC in decreasing order of frequency.

The ICA was present in all the cases, arising independently from SMA in almost all the cases. ICA was seen to arise as a CS with RCA in 2.00% of cases whereas Sonneland et al. reported such variation in 36.90% of cases [13]. The findings of the present study fully agreed with most of the researchers who reported ICA as the most consistent branch of SMA with classical description of the inferior most branch of SMA, supplying blood to the distal ileum, ileocecal valve, cecum, vermiform appendix, and the proximal one-third of ascending colon [25]. ICA and its branches are of major surgical and clinical significance. Special attention must be paid in the setting of colon cancer resection to sites of vessel anastomoses to include the right colic to ileocolic arteries for purposes of hemostasis [26].

Typically, cystic artery (CA) arises from the right hepatic artery. The other origins include SMA through its inferior pancreaticoduodenal branch [8]. It is not unusual for the CA to arise from any other source in the vicinity. Typically, accessory cystic artery denotes a vessel with aberrant origin supplying the gall bladder partially. 

In the present study, we found a very rare origin of the CA, which was taking origin from the SMA independently, supplied the gallbladder partially, and continued to the right lobe of liver. This kind of variant origin of CA, according to our knowledge is rarely reported in anatomical literature. Based on the presence of accessory arteries from SMA, such variant pattern can be classified as pattern IV of Gamo et al. classification [9]. Considering the fact that the diseases of the extra hepatic biliary apparatus often need surgical intervention, knowledge of the anatomical variations of the arterial supply of the gallbladder and liver is of great importance [20].

Inter-colic anastomosis, around the concave margin of large colon, forms a single arterial trunk and provides ample collateral circulation when required for planning of surgical intervention in this area [6,23]. It shows variations in terms of completeness and area of supply. In the present study, we found a fairly close association between SMA of pattern IIa and incomplete formation of marginal anastomosis, so right-sided colon supplied by pattern IIa SMA may be more prone to the gangrene formation after surgical intervention or due to occlusion of the feeding vessel (SMA). In-depth knowledge of the vascular anatomy of the colon and the associated pattern of collateral variation are necessary for surgeons to avoid both intra- and postoperative complications in surgical procedures involving the colon [27,28].

## Conclusions

Anatomic variations of the colon vasculature are complex and improper maneuvering during laparoscopic surgery can cause vascular complications. The MCA is responsible for major variations. Distinctive variations like MCA arising from CT or arising as CS with LCA are of outrageous importance for operating surgeons during right hemicolectomy and could potentially help in reducing vascular complications during minimally invasive procedures. Incomplete formations of inter-colic anastomosis are not an uncommon phenomenon and pattern IIa shows a fairly close association with such anomalous formations. The arc of Riolan formed by the branches of SMA and IMA is very crucial and incomplete anastomosis can affect its formation. Based on the study results, we propose a modification in Gamo’s classification of SMA anatomy to include the CS of MCA and LCA as pattern IId. Such modification is a simpler and better way to describe SMA anatomy based on its branching pattern, however, its success relies upon universal acceptance.
